# Hospital Festivals and Meetings

**Published:** 1895-07-27

**Authors:** 


### HOSPITAL FESTIVALS AND MEETINGS.

### THE ROYAL FREE HOSPITAL.—OPENING OF NEW FRONT BUILDINGS.

Monday last was a red-letter day in the annals of the Royal Free Hospital, when the new and handsome front building in the Gray's Inn Road, completing the work of reconstruction of the entire hospital, begun some forty-three years ago, was formally opened by T.R.H. the Prince and Princess of Wales. That something unusual was astir in the neighbourhood was abundantly evident from the streams of bunting floating gaily across the road outside the hospital, and a very large number of visitors had collected by half-past twelve o'clock. Inside the quadrangle was mounted a guard of honour of the Volunteer Medical Staff, by permission of Surgeon Lieut.-Colonel A. T. Norton; and the Prince and Princess, who were accompanied by the Princesses Victoria and Maud of Wales, and attended by Major-General Ellis, Major-General Stanley Clarke, and Lady Suffield, were received at the entrance by the Earl of Lathom, vice-president (in the absence of the President, the Marquis of Dufferin and Ava), and by the

Reception Committee, [and were thence conducted to the Milne Ward, and took up their positions on the central daïs. Here numbers of the students of the London School of Medicine for Women and their friends and visitors were assembled, and a bouquet of red and white carnations was presented to the Princess of Wales by one of the former.

The Earl of Lathom then read the following address: "May it please your Royal Highnesses,—The president, vice-presidents, Committee of Management, governors, and staff of the Royal Free Hospital beg to offer to your Royal Highnesses a most loyal and cordial welcome. The Royal Free Hospital was founded by the late Dr. William Marsden in the year 1828, and was the first hospital in the metropolis to admit the sick poor without letters of recommendation. Since its foundation its benefits have been extended to more than 2,000,000 patients, who claimed and obtained its aid by reason only of their poverty and suffering. It is the only general hospital in London which has opened its doors to female medical students, and for the past 18 years it has afforded means of clinical instruction to the students of the London School of Medicine for Women, which now has 150 students. In the year 1842 the hospital was removed to its present site. The north side was rebuilt in 1855 by the Freemasons of England as a memorial to his Royal Highness the late Duke of Sussex, and in 1877 the south side was erected and named the 'Victoria Wing,' in honour of her Most Gracious Majesty the Queen, who, shortly after her accession to the Throne, became its patron, and ordered that the institution should be called the Royal Free Hospital. The erection of the new front building completes the work of reconstruction commenced forty-three years since, and the hospital is now thoroughly equipped in every department for carrying on its beneficent work. May we be permitted to express our deep sense of gratitude for the benefits which your Royal Highnesses have conferred upon the hospital by the interest in its welfare which you have displayed, and more especially by the part which her Royal Highness the Princess of Wales has taken by inaugurating the new front building and graciously permitting it to be called the 'Alexandra building' in commemoration of this day's proceedings. We venture to express the hope that all who are interested in this great charity may be stimulated by this auspicious occasion to earnest effort in order to extend its usefulness and to increase its efficiency.—Signed, on behalf of the Governors, Dufferin and Ava (President), Gainsford Bruce (Chairman of the Committee), Charles Burt (Chairman of the Weekly Board), Conrad W. Thies (Secretary)."

The architect of the building, Mr. W. Harvey, was presented to their Royal Highnesses, and the Princess accepted from him a gold key of the new front entrance, and also a copy of the address inscribed on vellum. The Prince of Wales made a happy little speech, expressing the great pleasure it gave the Princess to "be here to-day and inaugurate the new wing which will complete this great hospital." His Royal Highness added that he did not forget that King George IV. and King William IV. were both patrons of the hospital, and also the Duchess of Kent and the Queen before she came to the Throne, and that Her Majesty had ever taken the liveliest interest in its prosperity.

Prayers having been said by the chaplain, purses were presented to the Princess by a number of children and others, some of the little ones exciting much amusement by their quaint little curtseys, and some being so tiny they had to be lifted to place their offerings, in their dainty blue purses, in the Princess's hands. Mrs. Wedgwood, mother of the matron of the hospital, and a daughter-in-law of Josiah Wedgwood, was presented to the Princess, and finally Mr. C. Burt, chairman of the weekly board, warmly thanked their Royal Highnesses for their presence, which had resulted in the collection of about £2,000 towards paying off the debt on the hospital. His tribute to the "Royal, gentle, and gracious Princess, whose nobility of character, kindness of heart, and tender sympathy for the sick and suffering poor were known all the world over," elicited a hearty response from all present.

On leaving the ward the Royal visitors were conducted to the lecture-room, where the lady students were assembled, and Mrs. Garrett-Anderson, M.D., the Dean, and Miss Julia Cork, Sub-Dean, with Mrs. Thorne, the secretary, were presented to them. In the operating theatre were gathered the nurses not on duty and the members of the medical and surgical staff, and their Royal Highnesses then visited three of the wards—the Elizabeth, Calthorpe, and Marsden Wards—after which they drove off amid much enthusiasm. It would have assuredly pleased the kindly Princess could she have been invisibly present in those same wards after her departure. The pleasure given by the interest shown by herself and the Prince in the patients was real and sincere, and those carnations cut by the Princess from her bouquet and distributed to the women and children, will be treasured for many a day as mementoes of "the dear, beautiful lady," who spoke to them words of sympathy and kindness.

Refreshments were provided for the visitors in an empty upstairs ward, and the hospital was generally open for inspection during the afternoon. It was fortunate that after a stormy morning the weather was bright and sunny, and the whole function went off without a hitch.

The cost of the new front building, with laundry and reconstruction of drainage, &c., has amounted to £30,000, towards which £24,000 has been raised. Every effort is therefore being made to pay off the remaining £6,000.

